# Molecular detection of *Babesia capreoli* and *Babesia venatorum* in wild Swedish roe deer, *Capreolus capreolus*

**DOI:** 10.1186/s13071-016-1503-8

**Published:** 2016-04-19

**Authors:** Martin O. Andersson, Ulrika A. Bergvall, Jan Chirico, Madeleine Christensson, Per-Eric Lindgren, Jonas Nordström, Petter Kjellander

**Affiliations:** Center for Ecology and Evolution in Microbial Model Systems (EEMiS), Linnaeus University, SE-391 82 Kalmar, Sweden; Molecular Ecology and Evolution Lab, Department of Biology, Lund University, Sölvegatan 37, SE-223 62 Lund, Sweden; Department of Zoology, Stockholm University, SE-106 91 Stockholm, Sweden; Grimsö Wildlife Research Station, Department of Ecology, Swedish University of Agricultural Sciences, SE-730 91 Riddarhyttan, Sweden; Department of Microbiology, National Veterinary Institute (SVA), Uppsala, Sweden; Department of Clinical and Experimental Medicine, Medical Microbiology, Linköping University, Linköping, Sweden; Medical Services, Microbiological Laboratory, County Hospital Ryhov, Jönköping, Sweden; Dalarna County Administration Board, SE-791 84 Falun, Sweden

## Abstract

**Background:**

The epidemiology of the zoonotic tick-transmitted parasite *Babesia* spp. and its occurrence in wild reservoir hosts in Sweden is unclear. In European deer, several parasite species, including *Babesia capreoli* and the zoonotic *B. venatorum* and *B. divergens* has been reported previously. The European roe deer, *Capreolus capreolus,* is an important and common part of the indigenous fauna in Europe, as well as an important host for *Ixodes ricinus* ticks, the vector of several *Babesia* spp. in Europe. Here, we aimed to investigate the occurrence of *Babesia* spp. in roe deer in Sweden.

**Findings:**

Roe deer (*n* = 77) were caught and sampled for blood. *Babesia* spp. was detected with a PCR assay targeting the 18S rRNA gene. The prevalence of *Babesia* spp. was 52 %, and two species were detected; *B. capreoli* and *B. venatorum* in 44 and 7.8 % of the individuals, respectively. Infection occurred both in summer and winter.

**Conclusions:**

We showed that roe deer in Sweden, close to the edge of their northern inland distributional range, are infected with *Babesia* spp. The occurrence of *B. venatorum* in roe deer imply that it is established in Sweden and the zoonotic implication of this finding should be regarded to a greater extent in future.

## Findings

### Background

The tick-transmitted intraerythrocytic parasite *Babesia* is maintained in zoonotic cycles between vertebrate hosts and tick vectors [1] and most zoonotic species are maintained in wildlife reservoirs. Various *Babesia* species have been detected in a wide range of different mammal species [1]. However, the occurrence in natural mammal hosts is still incompletely known for several zoonotic species [1]. The most prevalent zoonotic species, *Babesia microti*, is mainly reported from USA, and is maintained in various rodent reservoir hosts. In Europe, most human cases are attributed to the species *B. divergens* that is mainly associated with cattle. Moreover, also *B. venatorum* is known to infect humans in Europe [2, 3]. This species mainly utilizes roe deer as reservoir hosts [4]. Primarily *Babesia* spp. are of veterinary importance and cause severe economic losses in cattle and other domestic animals worldwide [5–8]. However since several species are also known to infect humans, babesiosis is considered as an emerging zoonosis in parts of the world [1, 9–11].

In European deer several *Babesia* spp. has been reported, including *B. capreoli, B. venatorum* and *B. divergens* [4, 12, 13]. There are some uncertainties as to what extent *B. divergens* is found in deer. Several samples have previously been sequenced and published on public databases as *B. divergens* or “*B. divergens*-like”. Recent re-sequencing of such samples have however convincingly identified them as the closely related *B. capreoli* [13]. However, actual *B. divergens* is found in red deer from Ireland [12]. *Babesia capreoli* is highly similar to *B. divergens* and the two species only differ at three nucleotide positions at the 18S rRNA gene (99.83 % nucleotide similarity) [13]. The two species are considered as indistinguishable based on morphological characteristics, sequencing is therefore necessary to identify these species [12, 13].

The roe deer (*Capreolus capreolus*) is the most common deer species in Sweden and occur in moderate to high population densities in the southern third of the country while population density gradually declines along a northern and western gradient to become completely absent in the north-western part of the mountain range [14]. *Babesia capreoli* has previously been reported from Swedish roe deer during the 1970’s based on microscopic findings in blood samples [15]. However, no confirmation with molecular methods of these findings has been performed.

In the present study we investigated the prevalence of *Babesia* and diversity of species in roe deer in two sites in south-central Sweden by using molecular tools.

### Sampling areas

Blood samples were taken from trapped roe deer at two different study sites, 150 km apart in southern Sweden; Bogesund (59°24′N, 18°12′E) is located at the inner reaches of the Stockholm Archipelago, surrounded by water and covered by highly productive mixed coniferous and deciduous forest and farmlands with high deer densities [16]. Grimsö Wildlife Research Area (59°60′N, 15°16′E) has a roe deer population with much lower density, and colder and longer winters due to its inland location. The area consists primarily of coniferous forest interspersed with bogs, mires and fens [17].

### Roe deer capture

A total of 48 adult and juvenile roe deer (> 7 months old) were captured in box-traps from January to March 2014 and blood samples were taken from the jugular vein. Captured deer were marked using ear-tags with unique ID numbers and colours to keep track of individuals. In addition to the adult and juvenile animals a total of 38 neonate roe deer fawns (1–40 days old) were sampled from May 15th to July 3rd during 2013. The blood was collected from the fawns’ tarsal vein.

### Ethical approval

The marking and handling of roe deer in this study were approved by the Ethical Committee on Animal Experiments, Uppsala, Sweden (Approval Dnr: C302/2012).

### Total nucleic acid extraction and PCR

Total nucleic acid (DNA as well as RNA) was extracted with the PAX gene Blood RNA kit (PreAnalytix, Qiagen/BD) following the manufacturer’s recommendations (without adding DNAse). Subsequently, cDNA was synthesized and the total DNA concentration was diluted to 10 ng/μl. PCR detection of *Babesia* spp. was carried out with the primers BJ1 5′-GTC TTG TAA TTG GAA TGA TGG-3′ and BN2 5′-TAG TTT ATG GTT AGG ACT ACG-3′ [18] with the cycling conditions as described in Casati et al. [18]. These primers amplify 411–452 bp of the 18S rRNA gene. PCR was performed in a GeneAmp® PCR System 9700 (Applied Biosystems). Sanger sequencing of the purified amplicons was performed and the obtained sequences were subjected to nucleotide BLAST searches on the NCBI database (http://www.ncbi.nlm.nih.gov).

### Results and discussion

We show with molecular methods that two *Babesia* spp. occur in wild roe deer in Sweden, *B. capreoli* and *B. venatorum.* This is, to the best of our knowledge, the first molecular detection of *Babesia* spp. in any wildlife species in Sweden. In total we obtained 86 blood samples from 77 individual roe deer. Nine individuals were re-captured on separate occasions. Most of them within a month of the first capture. Out of the recaptured individuals, two went from uninfected to infected, one individual lost the infection and two individuals went from being infected with one *Babesia* spp. to being infected with the heterologous *Babesia* spp., demonstrating the dynamic nature of *Babesia* infection in wild animals. Calculations of prevalence is based on the first capture of each individual. In total 52 % of the individuals (40 out of 77) were infected with *Babesia* spp. The prevalence of *B. capreoli* in the individuals were 44 % (34/77) and the prevalence of *B. venatorum* was 7.8 % (6/77). *Babesia capreoli* is the dominating *Babesia* species in Swedish roe deer in the investigated areas with a remarkably high prevalence, however, consistent with findings in central Europe that also reported high infection rates in roe deer [19]. Detailed information about the number of samples from animals caught in the different areas and the number of infections are presented in Table 1. The obtained sequences were all 100 % identical to the published *B. capreoli* sequence FJ944827 and clearly differed from *B. divergens* sequence U16370. *Babesia capreoli* and *B. divergens* differ from each other by only three nucleotides on the 18S rRNA gene, on positions 631, 663 and 1637 [13]. The two first positions are included in the DNA fragment amplified by the primers used in this study [16].

**Table 1:** *Babesia* spp. infection in roe deer individuals

Location	Grimsö	Bogesund	Total
Fawns			
*B. capreoli*	11 % (2/18)	27 % (4/15)	18 % (6/33)
*B. venatorum*	17 % (3/18)	13 % (2/15)	15 % (5/33)
Adult/juv.			
*B. capreoli*	87 % (13/15)	52 % (15/29)	64 % (28/44)
*B. venatorum*	6.7 % (1/15)	0	2.3 % (1/44)
Total *Babesia* spp.	58 % (19/33)	48 % (21/44)	52 % (40/77)

The *B. venatorum* sequences from the Swedish roe deer were identical to sequence KF724377 found in a human infection in China [20]*.* The sequences obtained in this study have been deposited in GenBank with the following accession numbers: *B. capreoli* KU145465 and *B. venatorum* KU145466.

*Babesia capreoli* is seemingly not able to infect cattle [13], and no reports of infections in humans have been published. This species is therefore not likely to be a threat to other species than the natural hosts [13]. Infection have been reported from several different deer species ([13] and references herein). Contrastingly, *B. venatorum* apparently has a broader host range and is also capable of infecting humans, it is also known to infect chamois (*Rupicapra rupicapra*) and ibex (*Capra ibex*) in the Alpine region [21], and has also been found in a captive reindeer (*Rangifer* sp.) in the Netherlands [22]. Several human cases of *B. venatorum* have been reported from Europe and more recently from China [2, 3, 23, 24] and the zoonotic potential of this species requires further investigation to correctly estimate risks for humans and perhaps domestic animals. *Babesia venatorum* has been reported from questing ticks in Norway [20] and a recent study on *Babesia* spp. in *Ixodes ricinus* in Sweden reported that 1 % of the investigated ticks were infected with *B. venatorum* [25]*.* Interestingly no ticks in that study were infected with *B. capreoli*, contrasting to the high prevalence found in roe deer in the present study. To better understand the importance of *Babesia* spp. as an infectious agent in Sweden there is a need to investigate the occurrence in several wild and domestic mammal species as well as in humans.
